# Factors associated with compliance with Infection Prevention and Control measures during the COVID-19 pandemic among healthcare workers in Kampala City, Uganda

**DOI:** 10.1371/journal.pone.0293732

**Published:** 2023-11-01

**Authors:** Mitima Jean-Marie Limenyande, Joyce Owens Kobusingye, Tonny Tindyebwa, Dorothy Akongo, John Bosco Isunju, David Musoke

**Affiliations:** 1 Makerere University School of Public Health, Kampala, Uganda; 2 Busoga Health Forum, Jinja, Uganda; University for Development Studies, GHANA

## Abstract

**Background:**

In the context of the COVID-19 pandemic that originated from China in December 2019 and spread around the world, Kampala City witnessed a high number of infections and deaths among healthcare workers (HCWs). This study assessed the level of compliance with Infection Prevention and Control (IPC) measures and its associated factors among HCWs during the COVID-19 pandemic, in Kampala City, Uganda.

**Methodology:**

A cross-sectional study was conducted in Nakawa Division, Kampala City, among 240 HCWs and used multistage sampling in government and private not-for-profit (PNFP) healthcare facilities. The outcome variable was self-reported IPC compliance which was composed of the use of masks, gloves, and hand hygiene. These were assessed using a 4-scale tool: always as recommended, most of the time, occasionally, and rarely. Only HCWs who responded “always as recommended” were considered compliant while the rest were considered non-compliant. Data was analyzed in STATA 14.0 using Modified Poisson regression to obtain factors associated with IPC compliance at 95% confidence interval (CI).

**Results:**

Forty-six (19.2%) respondents were compliant with all the three IPC measures, and this was associated with the presence of a COVID-19 patients’ ward in the healthcare facility (Adjusted Prevalence Ratio, APR: 2.51, 95%CI: 1.24–5.07). Factors associated with the use of masks were being of the Muslim religion (APR: 1.31, CI: 1.05–1.65), and working in a healthcare facility that has COVID-19 patients’ ward (APR: 1.29, CI: 1.06–1.59). Factors associated with the use of gloves were the age of the HCW, those above 40 years old being less complaint (APR: 0.47, CI: 0.24–0.93), working in the diagnosis department (APR: 2.08, CI: 1.17–3.70), and working in a healthcare facility that has COVID-19 patients’ ward (APR: 1.73, CI: 1.13–2.64). Factors associated with hand hygiene were working in a health center (HC) IV (PR: 1.7, CI: 1.26–2.30) or a HC II (PR: 1.68, CI: 1.28–2.21).

**Conclusion:**

Considering the elevated risk of disease transmission in health settings, IPC compliance was low; indicating an increased risk of COVID-19 infection among health care workers in Kampala City.

## Introduction

According to the World Health Organization, an estimated 80,000 to 180,000 healthcare workers (HCWs) died worldwide from COVID-19 between January 2020 and May 2021 [1]. This is because the risk of being infected among HCWs was found to be much higher compared to the general population [2]. Factors contributing to this increased risk included the fatigue from long hours and hard work without breaks. This resulted in mistakes while giving care such as the wrong usage of Personal Protective Equipment (PPE) or the use of the same PPE more than once [3]. Meanwhile, proper use of PPE could reduce the risk of getting infected by COVID-19 even after several contacts with positive cases [4]. The use of PPE needed to be rationalized to meet the demand of the pandemic whereby a shortage was expressed worldwide [5]. The protection of HCWs was critical due to the reduction of the labor workforce in healthcare facilities whereby those infected had to be quarantined or isolated for the required time [6, 7].

The COVID-19 pandemic had a severe impact on healthcare workers (HCWs) in Uganda, with 2,686 HCWs infected by the end of June 2021. The burden of infections among healthcare providers was unevenly distributed, with HCWs in Kampala and its five administrative divisions being the most affected. Almost 28 percent of infected HCWs were working in different healthcare facilities in the City. The increasing number of HCWs getting infected was worrying from a general point of view because they are important for the pandemic response. On the other hand, these HCWs could spread the infection to their colleagues and people in the community [8–10]. Different approaches were implemented to control the pandemic including a set of measures applied in healthcare facilities. At healthcare facility level, IPC guidelines and standard operating procedures (SOPs) were developed for the safety of HCWs [7, 10, 11]. Meanwhile, available information showed that compliance with IPC among HCWs in some regions remained low during the COVID-19 pandemic [12] and even in past epidemics [13], or depended on the perceived risk of getting infected in some instances [14].

Infection Prevention and Control (IPC) helps to prevent, at a low cost, deadly infectious diseases, and to prevent potential epidemics in healthcare units [15]. Among the IPC components, handwashing is considered to be one of the most cost-effective practices and is estimated to reduce up to 30% of healthcare-associated infections (HAI) although compliance remains low among HCWs [15, 16]. However, there are limitations to hand hygiene such as inadequate placement of handwashing stations to allow easy access while providing care. In addition, it is reported that washing hands many times can increase the risk of skin damage, exposing HCWs to get infections through the damaged skin [17]. On the other hand, gloves are effective in reducing hand contamination among HCWs during patient contact, and the use of masks was recommended as a safety measure in healthcare facilities given the mode of COVID-19 transmission [16, 18]. However, low compliance with IPC in health facilities may be due to inconsistencies and delays in the dissemination of the guidelines [19, 20]. Besides being unaware of the written guidelines, some employed personnel in healthcare settings have limited training, sometimes less than what is required even when they work at high-risk locations with regular contact with contagious patients [13, 21]. During epidemics, the availability of running water and enough stock of PPE are critical to support HCWs in implementing IPC practices but these resources are often lacking [13]. To facilitate IPC compliance and minimize the risk of infection during the process of donning and doffing, one approach is to treat a cohort of patients—that means treating patients with the same confirmed infection on the ward before changing the PPE [22]. However, this practice carries a risk of exposure if confirmed cases are confined with non-confirmed cases [13]. Another way to improve IPC compliance is to involve the patients in the IPC protocol by clearly explaining to them their role and the expected benefits. By improving the knowledge of patients on infection prevention, they will continuously monitor the practices of HCWs. This approach can increase cooperation among patients and reduce the risk of infection transmission to HCWs and other staff members [23, 24].

As the COVID-19 pandemic evolved, assessing the level of IPC compliance in different healthcare facilities in Kampala City became of great importance. While previous assessments focused on evaluating IPC compliance in COVID-19 treatment centers in Regional Referral facilities and various hospitals [25–28], there was limited knowledge concerning the extent of IPC compliance in other levels under the health care system, particularly in Kampala City, which was the epicenter of the COVID-19 outbreak in Uganda. For HCWs who were having a unique experience, the extent to which they followed the IPC guidelines for their safety remained an area of interest. With the scarcity of PPE and the explosive number of COVID-19 patients, it was relevant to understand what could determine whether or not a HCW would comply with IPC measures. This not only depended on individual factors but many other factors, from the institution they worked in, the community they served, and the government support. The aim of this study was, therefore, to identify the factors associated with IPC compliance among HCWs while providing care to patients during the COVID-19 pandemic.

## Methodology

### Study design and area

This was a cross-sectional study that included HCWs in healthcare facilities located in Nakawa Division, Kampala City. Nakawa Division was selected on the basis that it is mainly residential, has the biggest population compared to the other 4 divisions of the City, and registered more COVID-19 cases. The Division had a total of 287 healthcare facilities registered, and out of this total: 1) 7 healthcare facilities were owned by the government and were distributed as follows: 1 National Referral Hospital, 2 Hospitals, 1 Health Center (HC) III, and 3 HC II; 2)13 healthcare facilities were private not for profit (PNFP) and were distributed as follows: 1 HC IV, 3 HC III and 9 HC II; and 3) 267 were private for profit (PFP) and were distributed as follow: 4 Hospitals, 2 HC IV, 5 HC III, 256 HC II [29].

In the Ugandan healthcare system, a HC II targets 5,000 people and provides health services including preventive, promotive, outpatient, curative, and emergency delivery services. A HC III serves a larger community of 20,000 people and offers preventive, promotive, outpatient, curative, maternity, inpatient, and laboratory services. A HC IV extends its reach to accommodate 100,000 residents and builds upon the services provided by HC III by incorporating ultrasound examinations for obstetric cases, emergency and simple surgery including cesarean sections and lifesaving surgical operations, blood transfusion services, and mortuary. A hospital serves 500,000 people and in addition to the services offered at the community health centers, it provides services for general medical and surgical conditions, specialist services in Medicine, Surgery, Pediatrics, Community Medicine, and Obstetrics and Gynecology. It also provides in-service training, basic research, and internship [30].

### Sampling and study participants

The sample size was obtained using Kish Leslie formula for cross-sectional studies with a 95% confidence interval with a precision of 5%, a design effect of 1.5, and a 50% level of compliance. To account for potential non-response, the sample size was increased by 10% [12, 25, 31] and that gave a minimum of 635 respondents. A field assessment of the study site and the review of the literature [32, 33] revealed that the total population of HCWs in the eligible HFs in Nakawa Division was approximately 320. The sample size of 635 respondents was then adjusted using the formula $\frac{n}{1+\frac{n}{pop}}\;$[34], where n = 635 and pop = 320. The minimum sample size was thereafter 213 and probability proportional to size was applied for the different HF levels.

Purposive sampling technique was used to select healthcare facilities, and only government healthcare facilities and PNFP healthcare facilities were considered. Private for profit facilities were not included since they provide medical care for a limited group of people in the community who can afford it. For the same reason—of serving a specific part of the community, we excluded healthcare facilities used for police investigations and justice-related matters (1 HC III and 1 HC II), plus healthcare facilities serving the army and police personnel (1 National Referral Hospital, 1 Hospital, and 1 HC II). The remaining healthcare facilities that were eligible in the study were 1 hospital, 1 HC IV, 3 HC III, and 10 HC II.

One hundred fourteen (114) HCWs were selected in the hospital and 30 HCWs in the HC IV. In each of the two healthcare facilities, participants were selected from all departments using the list of HCWs that was generated from the daily schedules obtained from the departments. A random selection procedure was then done in Microsoft Excel, and the selected HCWs who were willing to be part of the study were then approached and interviewed thereafter. In the HC III, 45 HCWs from the 3 HC III took part in the study. Two of the 3 HC III had 10 and 14 HCWs who were eligible respondents, but only 8 HCWs responded to the tool in the first HC III and 9 in the second HC III. In the third HC III, 28 HCWs among those who were eligible agreed to be part of the study. Lastly, out of the 10 HC II, 7 were randomly selected and in each health facility, all eligible HCWs were approached for the interviews. The expected respondents were 28 with an average number of 4 HCWs per HC II but the final number obtained from HC II was 51 HCWs. Some HC II had more HCWs than the expected number.

HCWs registered in the selected healthcare facilities as nursing assistants, nurses, clinical officers, doctors, laboratory technicians, and interns were included, but their home residences were not necessarily located in Nakawa Division. In addition, social workers who were assisting medical workers with some tasks to reduce the workload were considered, that included taking vital signs, anthropometric measures, or other medical gestures. HCWs who assume only administrative tasks in the selected healthcare facilities were not included, plus hygienists and other support staff. Due to the nature of their position in the healthcare facility, they did not qualify for the outcome assessment.

### Data collection

The data was collected from 21^st^ March 2022 to 13^th^ May 2022 after a pretest in a HC IV among 10 respondents who were selected conveniently outside the study site. The outcome variable was IPC compliance and was assessed as a composite variable. It was composed of hand hygiene and the usage of PPE which included the use of masks and gloves. Each of the sub-variables was assessed using self-reported compliance and the questions were adapted from the WHO tool: “Risk assessment and management of exposure of health care workers in the context of COVID-19; Interim guidance” [35]. The response section in the tool has 4 scales for each PPE and for hand hygiene assessment, which are: always as recommended, most of the time, occasionally, and rarely. Always as recommended means the HCW is compliant more than 95% of the time, most of the time means 50% or more but not 100%, occasionally means 20% to under 50%, and rarely means less than 20% [35]. Only HCWs who responded “always as recommended” were considered compliant while the rest were considered non-compliant. For the overall compliance, HCWs who were always compliant to all the IPC measures were considered compliant and the rest non-compliant. During the assessment, “contact with the patient” was explained to the HCWs as moments like medical examination, lifting patients, or any other interaction involving physical contact during the process of providing care. This was intended to enlighten HCWs on moments they had to remember to minimize the risk of giving biased answers.

Handwashing is recommended during 5 moments: 1) before touching a patient, 2) before a procedure, 3) after a procedure or body fluid exposure risk, 4) after touching a patient and 5) after touching a patient’s surroundings. Out of the 5 moments recommended for handwashing only moments 1 and 4 were considered for this study since they apply to all HCWs in the study population regardless of the department where they work [15, 16]. If a HCW always washed hands for the 2 moments, he/she was considered compliant. For the purpose of this study, the specific duration for handwashing or the use of alcohol based hand rub, in order to be effective, was assumed to be constant for all the HCWs. Lastly, since both techniques of hand hygiene (handwashing and the use of alcohol based hand rub) are effective against COVID-19, if a HCW always applied any of the 2 approaches he was considered compliant with hand hygiene.

Compliance with the use of masks meant putting on a mask covering the mouth and the nose. In this study, if a HCW always put on any type of mask, he/she was considered compliant. Other remarks were not considered in the assessment of compliance with the use of masks, like the frequency of changing medical masks, and the use of different types of masks depending on their level of protection against COVID-19, including other approaches used to increase protection from COVID-19 like the use of double masks.

Regarding the use of gloves, a HCW was considered compliant if she/he always used gloves before any contact with the patient. Although it is required to use a new pair of gloves for each patient, reuse of gloves was not considered during the assessment of compliance in this study since the primary focus was HCWs’ safety. However, the reuse of gloves was captured and presented in the results since it is a risk of exposure to the patients being treated by the HCW.

Independent variables were assessed at healthcare facility level and individual level. Factors at the facility level were related to policies and organizational factors. Questions were administered to the IPC in-charges or the HCW delegated by the healthcare facility. Each of these variables was assessed using close-ended questions and for some variables like running water or the location of handwashing stations, confirmation was done through direct observation by the interviewers. HCWs were asked question on individual risk factors and socio-demographic characteristics. All these variables were assessed using close-ended questions.

### Data analysis

The process of data cleaning and organization was done using Microsoft Excel 2016, and STATA version 14.0 was used for the analysis. The age of the respondents and years of experience were collected as continuous variables and were thereafter transformed into categorical variables. The specific duty stations and the designation reported by HCWs were also grouped into broader categories where they belonged. Demographic characteristics and other independent variables were summarized using counts and percentages. Modified Poisson regression with robust variance was used to obtain the prevalence ratio (PR) values, and a p-value of less than 0.05 was considered significant. Binary logistic regression analysis was not used since the prevalence of IPC compliance was above 15%, the odds ratio (OR) obtained from logistic regression would overestimate the strength of association [36, 37]. For multivariable regression analysis, a p-value of 0.25 was used as the inclusion criteria in the model. For variables showing multicollinearity, meaning a correlation coefficient value greater or equal to 0.4, only one variable was left in the model. The modeling process used the backward technique and the final model included the adjusted Prevalence ratio (APR) for variables that showed an association with IPC compliance, with a significant p-value—that is less than 0.05. This modeling process was done successively for hand hygiene, the two PPE (mask and gloves), and lastly for the overall compliance with IPC.

### Ethical considerations

This study received ethical approval from Makerere University School of Public Health Higher Degrees Research and Ethics Committee, and Kampala Capital City Authority. Throughout the research process, we upheld the basic ethical principals in conducting this research. We obtained written informed consent from all the study participants and it was indicated to them that they were under no obligation to participate in the study and were free to withdraw at any time even after consenting.

## Results

### Characteristics of participants

Of the 240 participants involved in the study, 158 (65.8%) were female, 86 (35.8%) were from PNFP healthcare facilities, 51 (21.3%) from the HC II and 114 (47.5%) from the hospital, 46 (19.2%) were Muslim, 118 (49.2%) married, 123 (51.3%) aged below 30, and 39 (16.3%) had more than 10 years of work experience (Table 1).

**Table 1:** Socio-demographic characteristic of study participants.

Variables	Number (N = 240)	Percentage (%)
**Sex**		
Female	158	65.8
Male	82	34.2
**Age (years)**		
<30	123	51.3
30–39	75	31.3
≥ 40	42	17.5
**Marital Status**		
Married	118	49.2
Not married	122	50.8
**Religion**		
Catholic	86	35.8
Muslim	46	19.2
Others	108	45.0
**Designation**		
Doctor	26	10.8
Clinical officer	48	20.0
Nurse	80	33.3
Lab technician	25	10.4
Intern	61	25.4
**Years of experience**		
≤ 3	118	49.2
4–10	83	34.6
> 10	39	16.3
**Facility ownership**		
Government	154	64.2
PNFP	86	35.8
**Facility level**		
Hospital	114	47.5
HC IV	30	12.5
HC III	45	18.8
HC II	51	21.3
**Department**		
Out Patient Department (OPD)	43	17.9
Medical	48	20.0
Surgery	35	14.6
Pediatric	33	13.8
Obstetrics—Gynecology	41	17.1
Laboratory and others	40	16.7

### Level of compliance with IPC measures

#### Compliance with the use of masks

Of the 240 healthcare workers who took part in the study, 163 (67.9%) were compliant with the use of masks and this had the highest level of compliance among the IPC measures that were assessed. HCWs who were not married and were compliant with the use of masks represented 73.8%. Considering the religion of respondents, Muslims were more compliant; 82.6% of them reported putting on face masks always while interacting with patients. HCWs from HC II were the least compliant with only 51.7% using masks always. Healthcare facilities that had COVID-19 wards had more compliant HCWs (75.7%) to the use of masks than those that did not have (56.3%), (Table 2).

**Table 2:** Factors associated with the use of mask and the use of gloves.

Variables	Compliance with the use of mask			Compliance with the use of gloves		
	N = 240	UPR (95% CI)	APR (95% CI)	N = 240	UPR (95% CI)	APR (95% CI)
	n(%)			n(%)		
**Sex**						
Female	106(67.1)	1.0		50(31.7)	1.0	
Male	57(69.5)	1.03(0.86–1.21)	- -	31(37.8)	1.19(0.83–1.71)	- -
**Age (years)**						
<30	83(67.5)	1.0		47(38.2)	1.0	1.0
30–39	51(68.0)	1.00(0.82–1.22)	- -	27(36.0)	0.94(0.64–1.34)	0.96(0.65–1.40)
≥ 40	29(69.1)	1.02(0.59–2.29)	- -	7(16.7)	0.43(0.21–0.89) *	0.47(0.24–0.93) *
**Marital status**						
Married	73(61.9)	1.0		37(31.4)	1.0	
Not married	90(73.8)	1.19(0.99–1.42)	- -	44(36.1)	1.15(0.80–1.64)	- -
**Religion**						
Catholic	50(58.1)	1.0	1.0	28(32.6)	1.0	
Muslim	38(82.6)	1.42(1.13–1.77) **	1.31(1.05–1.65) *	21(45.7)	1.40 (0.90–2.17)	- -
Others	75(69.4)	1.19(0.95–1.48)	1.19(0.96–1.48)	32(29.6)	0.91(0.59–1.38)	- -
**Years of experience**						
≤ 3	84(71.2)	1.0		42(35.6)	1.0	
4–10	52(62.7)	0.88(0.71–1.07)	- -	29(34.9)	0.98(0.53–1.43)	- -
> 10	27(69.2)	0.97(0.76–1.23)	- -	10(25.6)	0.72(0.40–1.29)	- -
**Facility ownership**						
Government	116(75.3)	1.0	- -	53(43.4)	1.0	
PNFP	47(54.7)	0.72(0.58–0.89) **	- -	28(32.6)	0.94(0.65–1.37)	- -
**Facility level**						
Hospital	87(76.3)	1.0		44(38.6)	1.0	
HC IV	22(73.3)	0.96(0.75–1.22)	―	15(50.0)	1.29(0.84–1.98)	―
HC III	26(57.8)	0.75(0.57–0.99) *	―	7(15.6)	0.40(0.19–0.82) *	―
HC II	28(54.9)	0.71(0.54–0.94) *	―	15(29.4)	0.76(0.46–1.23)	―
**Department**						
OPD	26(60.5)	1.0		11(25.6)	1.0	1.0
Medical	32(66.7)	1.10(0.80–1.50)	- -	10(20.8)	0.81(0.38–1.72)	0.91(0.44–1.87)
Surgery	26(74.3)	1.22(0.90–1.67)	- -	17(48.6)	1.89(1.02–3.51) *	1.59(0.86–2.94)
Pediatric	25(75.8)	1.25(0.91–1.70)	- -	7(21.2)	0.82(0.36–1.90)	0.68(0.29–1.58)
Obstetrics—Gynecology	27(65.9)	1.08(0.78–1.51)	- -	16(39.0)	1.52(0.80–2.88)	1.42(0.75–2.70)
Laboratory and others	27(67.5)	1.11(0.80–1.54)	- -	20(50.0)	1.95(1.07–3.55) *	2.08(1.17–3.70) *
**Designation**						
Doctor	18(69.2)	1.0		13(50.0)	1.0	
Clinical officer	30(62.5)	0.90(0.64–1.26)	―	9(18.8)	0.37(0.18–0.75) **	―
Nurse	57(71.3)	1.02 (0.76–1.37)	―	22(27.5)	0.55(0.32–0.92) *	―
Lab	16(64.0)	0.92 (0.62–1.36)	―	16(64.0)	1.28(0.78–2.07)	―
Intern	42(68.9)	0.99 (0.73–1.35)	―	21(34.4)	0.68(0.40–1.15)	―
**HCW received IPC training**						
No	31(62.0)	1.0		15(30.0)	1.0	
Yes	132(69.5)	1.12 (0.88–1.42)	- -	66(34.7)	1.15(0.72–1.84)	- -
**Last IPC training**						
Never had	27(58.7)	- -	- -	14(30.4)	- -	- -
This year	124(70.9)	- -	- -	64(36.6)	- -	- -
More than one year	12(63.2)	- -	- -	3(15.8)	- -	- -
**HCW had a health risk for severe COVID-19**						
No	138(68.7)	1.0		70(34.8)	1.0	
Yes	25(64.1)	0.93 (0.72–1.20)	- -	11(28.2)	0.80(0.47–1.38)	- -
**HCW had a family member with a health risk for severe COVID-19**						
No	98(67.1)	1.0		52(35.6)	1.0	
Yes	65(69.2)	1.03 (0.86–1.22)	- -	29(30.9)	0.86(0.45–1.59)	- -
**HCW had been infected with COVID-19**						
No	112(66.7)	1.0		55(32.7)	1.0	
Yes	51(70.8)	1.06(0.88–1.27)		26(36.1)	1.10(0.75–1.60)	- -
**HCW had highly suspicious symptoms of COVID-19**						
No	51(68.0)	1.0	- -	25(33.3)	1.0	- -
Yes	112(67.9)	0.99 (0.82–1.20)	- -	56(33.9)	1.01(0.69–1.49)	- -
**HCW worked in more than one HF**						
No	132(68.4)	1.0		63(3.6)	1.0	
Yes	31(66.0)	0.96(0.76–1.21)	- -	18(38.3)	1.17(0.77–1.77)	- -
**HCW was vaccinated against COVID-19**						
No	18(72.0)	1.0		8(32.0)	1.0	
Yes	145(67.4)	0.93 (0.72–1.21)	- -	73(34.0)	1.06(0.58–1.93)	- -
**HF had an IPC team**						
No	6(40.0)	- -		3(20.0)	- -	
Yes	157(69.8)	- -	- -	78(34.7)	- -	- -
**HF had a COVID-19 patients’ ward**						
No	54(56.3)	1.0	1.0	22(22.9)	1.0	1.0
Yes	109(75.7)	1.34(1.10–1.64) **	1.29(1.06–1.59) *	59(41.0)	1.78(1.17–2.71) **	1.73(1.13–2.64) *
**Single-use of gloves per patient**						
No				13(13.1)	1.0	
Yes				86(48.2)	3.67(2.14–6.27) ***	- -
**Use of gloves compliance**						
No	96(60.4)	1.0				
Yes	67(82.7)	1.36 (1.16–1.60) ***				
**Hand hygiene compliance**						
No	65(58.0)	1.0				
Yes	98(76.6)	1.31 (1.09–1.58) **				

Key: n = number of compliant HCWs, UPR: Unadjusted Prevalence ratio, APR: Adjusted Prevalence Ratio,

*: p<0.05,

**: p<0.01,

***: p<0.001,

- -: p-value not significant (p-value>0.05),

―:variable removed from the model due to collinearity

#### Compliance with the use of gloves

The number of HCWs who were compliant with the use of gloves was 81 (33.8%), and this was the IPC measure with the least level of compliance compared to the two others (mask and hand hygiene). All the age brackets had a level of compliance with the use of gloves below 50% and HCWs aged above 40 years had the lowest level of compliance with 16.7%. The percentage of HCW in surgical wards and laboratories who were compliant represented 48.6% and 50% respectively while those in the pediatric ward represented 21.2%. Single-use of gloves per patient was reported among 141 (58.8%) HCWs out of the 240 respondents (Table 2).

#### Compliance with hand hygiene

The level of compliance with hand hygiene was 53.3% (128) among respondents. Of the 128 respondents, 73 (30.4%) reported using alcohol based hand rub always as a means of hand hygiene, 21 (8.8%) reported washing hands always, and 34 (14.2%) both hand washing and hand sanitizing. In healthcare facilities where hand washing stations are easily accessible, HCWs were compliant with hand hygiene at 61.7% and when soap was provided, the compliance level was 68.2% (Table 3).

**Table 3:** Factors associated with hand hygiene and overall IPC compliance.

Variables	Hand hygiene compliance			Overall IPC compliance		
	N = 240	UPR (95% CI)	APR (95% CI)	N = 240	UPR (95% CI)	APR (95% CI)
	n(%)			n(%)		
**Sex**						
Female	79(50.0)	1.0		26(16.5)	1.0	
Male	49(59.8)	1.19(0.94–1.51)	- -	20(24.4)	1.48(0.88–2.49)	- -
**Age (years)**						
<30	67(54.5)	1.0		27(22.0)	1.0	1.0
30–39	39(52.0)	0.95(0.72–1.25)	- -	16(21.3)	0.97(0.56–1.68)	1.07 (0.62–1.85)
≥ 40	22(52.4)	0.96(0.69–1.33)	- -	3(7.2)	0.32(0.10–1.02)	0.41(0.13–1.30)
**Marital status**						
Married	61(51.7)	1.0		21(17.8)	1.0	
Not married	67(54.9)	1.06(0.83–1.34)	- -	25(20.5)	1.15(0.68–1.94)	- -
**Religion**						
Catholic	42(48.8)	1.0		18(20.9)	1.0	
Muslim	23(50.0)	1.02(0.71–1.47)	- -	15(32.6)	1.55(0.86–2.79)	- -
Others	63(58.3)	1.19(0.91–1.56)	- -	13(12.0)	0.57(0.29–1.10)	- -
**Years of experience**						
≤ 3	61(51.7)	1.0		23(19.5)	1.0	
4–10	47(56.6)	1.09(0.84–1.41)	- -	18(21.7)	1.11(0.64–1.92)	- -
> 10	20(51.3)	0.99(0.69–1.41)	- -	5(12.8)	0.65(0.26–1.61)	- -
**Facility ownership**						
Government	78(50.7)	1.0		32(20.8)	1.0	
PNFP	50(58.1)	1.14(0.90–1.45)	- -	14(16.3)	0.78(0.44–1.38)	- -
**Facility level**						
Hospital	49(43.0)	1.0		26(22.8)	1.0	
HC IV	22(73.3)	1.70(1.26–2.30) **	―	11(36.7)	1.60(0.89–2.87)	―
HC III	20(44.4)	1.03 (0.70–1.52)	―	5(11.1)	0.48 (0.19–1.19)	―
HC II	37(72.6)	1.68(1.28–2.21) ***	―	4(7.8)	0.34 (0.12–0.93) *	―
**Department**						
OPD	26(69.5)	1.0		8(18.6)	1.0	
Medical	23(47.9)	0.79(0.54–1.16)	- -	5(10.4)	0.55(0.19–1.58)	- -
Surgery	18(51.4)	0.85(0.56–1.27)	- -	13(37.1)	1.99(0.93–4.27)	- -
Pediatric	17(51.5)	0.85(0.56–1.28)	- -	6(18.2)	0.97(0.37–2.54)	- -
Obstetrics—Gynecology	20(48.8)	0.80(0.54–1.19)	- -	5(12.2)	0.65(0.23–1.84)	- -
Laboratory and others	24(60.0)	0.99(0.69–1.40)	- -	9(22.5)	1.20(0.51–2.83)	- -
**Designation**						
Doctor	16(61.5)	1.0		9(34.6)	1.0	
Clinical officer	33(68.8)	1.11(0.77–1.60)	―	6(12.5)	0.36(0.14–0.90) *	―
Nurse	44(55.0)	0.89(0.62–1.28)	―	15(18.8)	0.54(0.16–1.16)	―
Lab	11(44.0)	0.71(0.41–1.22)	―	6(24.0)	0.69(0.28–1.66)	―
Intern	24(39.3)	0.63(0.41–0.98) *	―	10(16.4)	0.47(0.21–1.02)	―
**HCW received IPC training**						
No	29(58.0)	1.0		10(20.0)	1.0	
Yes	99(52.1)	0.89(0.68–1.18)	- -	36(19.0)	0.94(0.50–1.77)	- -
**Last IPC training**						
Never had	27(58.7)	1.0		9(19.6)	1.0	
This year	90(51.4)	0.87 (0.66–1.16)	- -	36(20.6)	1.05(0.54–2.02)	- -
More than one year	11(58.0)	0.98(0.62–1.55)	- -	1(5.3)	0.26(0.3–1.98)	- -
**HCW had a health risk for severe COVID-19**						
No	107(53.2)	1.0		41(20.4)	1.0	
Yes	21(53.9)	1.01(0.73–1.39)	- -	5(12.8)	0.62(0.26–1.49)	- -
**HCW had a family member with a health risk for severe COVID-19**						
No	79(54.1)	1.0		30(20.6)	1.0	
Yes	49(52.1)	0.96(0.75–1.23)	- -	16(17.0)	0.82(0.47–1.43)	- -
**HCW had been infected with COVID-19**						
No	94(56)	1.0		30(17.7)	1.0	
Yes	34(47.2)	0.84(0.63–1.11)	- -	16(22.2)	1.24(0.72–2.13)	- -
**HCW had highly suspicious symptoms of COVID-19**						
No	43(57.3)	1.0		13(17.3)	1.0	
Yes	85(51.5)	0.89(0.70–1.14)	- -	33(20.0)	1.15(0.64–2.06)	- -
**HCW worked in more than one HF**						
No	103(53.4)	1.0		34(17.6)	1.0	
Yes	25(53.2)	0.99(0.73–1.34)	- -	12(25.5)	1.44(0.1–2.58)	- -
**HCW was vaccinated against COVID-19**						
No	13(52.0)	1.0		2(8.0)		
Yes	115(53.5)	1.02(0.69–1.53)	- -	44(20.5)	2.55(0.65–9.94)	- -
**HF had an IPC team**						
No	29(58.0)	1.0		1(6.7)		
Yes	99(52.1)	1.64(0.79–3.39)	- -	45(20.0)	- -	- -
**HF had a COVID-19 patients’ ward**						
No	57(59.4)	1.0		9(9.4)	1.0	1.0
Yes	71(49.3)	0.83(0.65–1.04)	- -	37(25.7)	2.74(1.38–5.42) **	2.51 (1.24–5.07) *
**Hand washing stations were at the right location**						
No	62(46.6)	1.0		27(20.3)	1.0	
Yes	66(61.7)	1.32(1.04–1.67) *	- -	19(17.7)	0.84(0.44–1.62)	- -
**Soap was available at the handwashing stations**						
No	98(50.0)	1.0		35(17.9)	1.0	
Yes	30(68.2)	1.36(1.06–1.74) *	- -	11(25.0)	1.53(0.70–3.32)	- -

Key: n = number of compliant HCWs, UPR: Unadjusted Prevalence ratio, APR: Adjusted Prevalence Ratio,

*: p<0.05,

**: p<0.01,

***: p<0.001,

- -: p-value not significant (p-value>0.05),

―:variable removed from the model due to collinearity

#### Overall compliance

Only 46 (19.2%) respondents were compliant with all the IPC measures that were assessed in the study. The compliance was at 16.5% among females, 7.2% among HCWs aged 40 years and above, and 17% among those who are married. Only 6.7% of HCWs in HC II were compliant with all the IPC measures. Among those who reported that they had received IPC training, 19% were compliant whereas for those who reported that they have never received any COVID-19-related IPC training, 20% were compliant (Table 3).

### Factors associated with IPC compliance

#### Compliance with the use of masks

Factors that were found to be associated with the use of masks included the religion of the respondent and whether or not the healthcare facilities had a COVID-19 ward or admitted COVID-19 patients. The proportion of Muslims who were compliant with the use of masks was nearly 1.3 times the proportion of Catholics (APR: 1.31, 95% CI: 1.05–1.65). HCWs that worked in healthcare facilities that admitted or had a COVID-19 ward were 1.3 times (APR: 1.29, 95% CI: 1.06–1.59) the proportion of those working in facilities that did not have a COVID-19 ward nor admitted COVID-19 patients. It was also noted that HCWs who were compliant with the use of masks had also a higher proportion of them being compliant with the use of gloves (PR: 1.36, 95% CI: 1.16–1.60) and compliant with hand hygiene (PR: 1.31, 95% CI: 1.09–1.58) (Table 2).

#### Compliance with the use of gloves

Factors associated with the use of gloves included the age of the HCW as the prevalence of the use of gloves among those aged above 40 years old was less compared to those aged below 40 (APR: 0.47, 95% CI:0.24–0.93). Another factor associated with the use of gloves was the department where the HCW worked. The proportion of those working in the laboratory and other medical diagnostic departments was 2 times more (APR: 2.08, 95% CI: 1.17–3.70) as compared to the proportion of HCWs who worked in the OPD department and who were compliant with the use of gloves. Lastly, we found that being in a healthcare facility that admitted COVID-19 patients or had a COVID-19 ward was associated with a higher chance of using gloves. There was a 1.7-fold increase in the prevalence of using gloves (APR: 1.73, 95% CI: 1.13–2.64) in HCWs from healthcare facilities with COVID-19 wards compared to those in healthcare facilities without a COVID-19 ward (Table 2).

### Factors associated with compliance to hand hygiene

The prevalence of hand hygiene compliance was found to be higher in the HC IV than in the hospital (PR: 1.70, 95% CI: 1.26–2.30), and higher in HC II than in the hospital (PR: 1.68, 95% CI: 1.28–2.21). The location of the handwashing station or the provision of soap for hand washing were also associated with a high prevalence of hand hygiene compliance. The proportion of HCWs who worked in healthcare facilities with easy access to hand washing stations (PR: 1.32, 95% CI: 1.04–1.67), and those working where soap was always provided (PR: 1.36, 95% CI: 1.06–1.74) were more compared to the proportion of those who worked in healthcare facilities that did not provide soap always or hand washing stations were not easily accessible (Table 3).

### Factors associated with the overall IPC compliance

Considering the 3 components of IPC that were assessed, the presence of a COVID-19 ward in the healthcare facility was found to be associated with IPC compliance. The level of compliance to all the three IPC measures among HCWs in healthcare facilities with a COVID-19 patients’ ward was 2.5 times higher compared to those who worked in healthcare facilities that did not have a COVID-19 ward (APR: 2.51, 95% CI: 1.24–5.07) (Table 3).

## Discussion

IPC compliance in healthcare facilities has continuously increased interest in recent years and further amplified during the COVID-19 pandemic due to its impact on the transmission of the virus among healthcare workers. Understanding HCWs’ behavior is crucial for effective IPC implementation in healthcare settings, and this study identified key factors that can be targeted for interventions. This study revealed that Healthcare workers exhibited a generally low level of compliance with IPC measures, which has been a long-standing challenge in health services delivery [16]. The findings of this study showed that compliance with hand hygiene and the use of gloves remained poor, while the use of masks, which became more prominent during the pandemic, had relatively better compliance compared to the two other IPC measures. In the high-risk environment of COVID-19, some HCWs recognized the importance of protecting themselves by wearing personal protective equipment (PPE) and adhering to hand hygiene practices [38, 39]. Therefore, this is then an opportunity to explore if an IPC behaviour change is to be obtained from HCWs whereby they should be informed about the risk they face regarding COVID-19. Secondly, findings from this study could be used to improve IPC compliance during routine care by showing HCWs the risks and benefits of every IPC measure they skip though it is required. A study conducted in Germany also found that compliance with PPE use was higher in COVID-19 wards, but even in those wards, not all HCWs were compliant [14]. This finding upholds the fact that the high risk of contracting the disease was an important factor that triggered HCWs to act in the right way for their safety [40, 41].

The use of face masks was associated with the religion of respondents and the presence of a COVID-19 patients’ ward in the healthcare facility. Muslims were more likely to be compliant with the use of masks than other religions. This observation aligns with previous studies that have highlighted the impact of religion and culture on IPC-related behavior [16, 38, 42]. One possible explanation for this finding is that some Muslims are used to covering themselves using the hijab, which makes them more comfortable wearing masks. There is a strong argument that this practice among Muslim women may have played a role in reducing the transmission of COVID-19 infection in countries where the majority of the population is Muslim [43]. However, this issue may need to be explored further to better understand and confirm the supporting explanations for the higher compliance among Muslims in this study. Additionally, it is important to bear in mind that the cultural norm of covering oneself basically applies to female Muslims in the specific City where our study was conducted, and thus more likely to fulfill the assumption of feeling less uncomfortable in facemasks.

The use of face masks among healthcare workers was found to be associated with the presence of a COVID-19 ward in the healthcare facility. This finding is consistent with previous studies conducted in Germany, China, and Ghana that a high level of compliance with the use of masks had a linkage with the presence of COVID-19 patients in a ward of a healthcare facility [14, 44, 45]. The same tendency was found in Middle East countries during the MERS pandemic in 2012, where substantial compliance with the use of masks was noticed among HCWs [42]. Meanwhile, the level of compliance with the use of masks in these studies was generally higher compared to our findings due to the way compliance was assessed, or the difference in the timeline of the pandemic; some studies were conducted at the early stage of the pandemic and others during the peak or post peak of the pandemic [46]. However, regardless of these disparities, all studies suggest that the presence of life-threatening infections in healthcare facilities triggers improvement in adherence to IPC measures. The need for self-protection emerged as a crucial motivator for HCWs to wear masks during the COVID-19 pandemic, as reported by the findings of our study.

The use of gloves in our study was found to be significantly lower among HCWs aged 40 years and above. In a previous study, their risk of developing severe symptoms of COVID-19 was higher than in younger HCWs [47]. In Nigeria, HCWs aged below 45 years were reported to be having a better knowledge of PPE usage [48]. Compliance with PPE usage, especially gloves, has shown serious implementation challenges as a practice in health service delivery [16]. Evidence from previous studies conducted in China and Kenya showed that the use of gloves was generally inadequate among HCWs [45, 49]. Although age is an important factor, the level of compliance with the usage of gloves as reported by different studies differs according to the approaches used to assess the outcome [44, 45, 47]. Therefore, age is an important factor to consider in any activity that aims to improve the use of gloves in health service delivery.

The use of gloves had the lowest level of compliance among HCWs compared to the two other IPC measures (use of mask and hand hygiene). It has been reported previously that the inadequacy of the use of gloves is sometimes a result of a culture adopted by HCWs in a healthcare facility [50]. Poor compliance with the use of gloves in our study may then be justified by the fact that HCWs who were aged above 40 years could be more likely to be affected by the routine of their work. Given that many of them have been working in a health system where the scarcity of gloves and other PPE was frequent, and they were used to attending to patients without wearing gloves. With time, this absence of PPE may have stayed as a norm in healthcare facilities with minimal attention given to it. Nevertheless, the shortage of PPE was forecast at the beginning of the pandemic, and this was one of the fears expressed by HCWs—the fear of not accessing PPE for their safety [5, 51]. On the other hand, the health system made an effort to provide PPE during the COVID-19 pandemic which might have influenced younger HCWs to comply with the use of gloves. Their assured access to PPE while providing care to potential COVID-19 patients played a big role. It is, therefore, important to consider the provision of gloves at all times to enhance usage compliance.

Washing hands as a protective measure against COVID-19 differed across healthcare facility levels. This practice was significantly higher in HC IVs and HCIIs compared to the hospital. The difference in IPC compliance across health system levels was also noticed in Ethiopia [12]. However, in our findings, the level of compliance with hand hygiene was generally low, and it was reported that most HCWs prefer using hand rub sanitizer to washing hands. Hand sanitizing was highly promoted during the COVID-19 pandemic and has been recommended for hand hygiene given the challenges of accessing hand washing stations during routine care in healthcare facilities [44, 45, 52]. Hand hygiene using alcohol based hand rub or hand washing using water and soap has been well-differentiated in recommendations by WHO [16], and this can therefore be promoted to make it part of every day’s behavior of HCWs. In our study, most HCWs reported using alcohol based hand rub than washing hands. HCWs need sensitization on the advantages of each of the two approaches used for hand hygiene: when should hands be washed and when alcohol based hand rub should be performed. It should be made clear that washing hands is required in the theatre before aseptic interventions, and in routine care. In routine care, hand washing is recommended when hands are soiled or when they have been in contact with blood, fluid, etc. For the rest of the time, when hands are clean, hand sanitizing remains beneficial and can save time [15, 16, 53, 54].

Similar to our study, a low level of compliance with hand washing among HCWs was also found in Italy during the COVID-19 pandemic, following a continuous assessment over six years. It was observed that the level of compliance with hand hygiene improved from 45% to 66% following continuous monitoring [53]. The reason for the poor compliance, despite the high risk of contagion in the COVID-19 pandemic, was attributed to the inflow of patients that increased in their region compared to the availability HCWs [53, 55]. The risk of being infected was found to be a motivator for HCWs who still followed the guidelines on hand hygiene and other IPC measures. There is, consequently, a clear need to emphasize the training of HCWs who generally expressed a lack of adequate knowledge on IPC and who rely on the perceived risk of being infected.

One strength of this study is that respondents were selected from different levels of the health system using probability sampling, enhancing the representativeness of our findings. However, a few limitations exist: First; the data collected relied heavily on self-reported compliance from HCWs, which may have been subject to social desirability bias, leading to potential overestimation of compliance levels. To address this limitation, we took measures to clearly communicate the study’s purpose to the respondents and encourage honest reporting. Secondly, we excluded cleaners and support staff from our assessment since they were not part of the outcome assessment criteria. Future studies should consider incorporating them to gain a more comprehensive understanding of IPC practices in these settings and capture the perspectives of all relevant healthcare personnel.

## Conclusion

The level of compliance with IPC measures among HCWs in Nakawa Division, Kampala City, was low during the COVID-19 pandemic despite the high risk of being infected. The use of gloves had the least level of compliance compared to the use of face masks and hand hygiene. The main factor associated with HCWs’ compliance with IPC measures during the COVID-19 pandemic in Kampala City was the presence of a COVID-19 ward in the healthcare facility. Compliance with the use of gloves was associated with the presence of a COVID-19 ward in the healthcare facility, the age of the HCW, and the department a HCW worked in. Compliance to IPC measures among HCWs during pandemics could be enhanced by providing enough PPE, ensuring hand hygiene facilities are available and accessible, always providing alcohol based hand rub, and improving the knowledge of HCWs on IPC through capacity building. These interventions can contribute to improving compliance with IPC measures and ultimately enhance the safety and well-being of HCWs and patients during infectious disease outbreaks.

## Supporting information

S1 Dataset (XLSX)
